# Concise Review: Mesenchymal Stem Cell Treatment of the Complications of Diabetes Mellitus

**DOI:** 10.1002/stem.556

**Published:** 2010-11-09

**Authors:** Vladislav Volarevic, Nebojsa Arsenijevic, Miodrag L Lukic, Miodrag Stojkovic

**Affiliations:** a Centre for Molecular Medicine, Faculty of Medicine, University of KragujevacKragujevac, Serbia; b Department of Microbiology and Immunology, Faculty of Medicine and Health Sciences, UAE UniversityAl Ain, United Arab Emirates; c Spebo MedicalLeskovac, Serbia

**Keywords:** Mesenchymal stem cell, Cardiomyopathy, Nephropathy, Neuropathy, Wound, Diabetes mellitus

## Abstract

Mesenchymal stem cells (MSCs) are multipotent, self-renewing cells that can be found in almost all postnatal organs and tissues. The main functional characteristics of MSCs are their immunomodulatory ability, capacity for self-renewal, and differentiation into mesodermal tissues. The ability of MSCs to differentiate into several cell types, including muscle, brain, vascular, skin, cartilage, and bone cells, makes them attractive as therapeutic agents for a number of diseases including complications of diabetes mellitus. We review here the potential of MSCs as new therapeutic agents in the treatment of diabetic cardiomyopathy, diabetic nephropathy, diabetic polyneuropathy, diabetic retinopathy, and diabetic wounds. Also, in this review we discuss the current limitations for MSCs therapy in humans. Stem Cells 2011;29:5–10

## INTRODUCTION

Mesenchymal stem cells (MSCs), also known as multipotent mesenchymal stromal cells, are self-renewing cells that can be found in almost all postnatal organs and tissues [1]. MSCs are most frequently isolated from bone marrow but can generally be derived from any organ [2]. Depending on their intended purpose, experimental or therapeutic use, MSCs can be isolated from adipose tissue, umbilical cord blood, compact bone, and other tissues [2]. MSCs show variable expression levels of several molecules: CD105 (SH2), CD73 (SH3/4), stromal antigen 1, CD44, CD90, CD166 (vascular cell adhesion molecule), CD54/CD102 (intracellular adhesion molecule), and CD49 (very late antigen) [3, 4]. Conversely MSCs lack the expression of surface markers characteristic for hematopoietic cells (CD14, CD45, and CD11a/lymphocyte function-associated antigen 1 (LFA-1)), erythrocytes (glycophorin A), and platelet and endothelial cell markers (CD31) [5] (Supporting Information Fig. 1).

The main functional characteristics of MSCs are their immunomodulatory ability, capacity for self-renewal, and differentiation into tissues of mesodermal origin [6, 7]. Through production of soluble factors, MSCs can alter the secretion profile of dendritic cells (DCs) resulting in increased production of anti-inflammatory cytokine interleukin (IL)-10 and decreased production of interferon-gamma (IFN-γ) and IL-12 [6–8]. MSCs can inhibit T-cell proliferation by engagement of the inhibitory molecule programmed death 1(PD-1) to its ligands PD-L1 and PD-L2, thereby producing soluble factors that suppress T-cell proliferation (such as TGF-β or IL-10) and through interacting with DCs [7, 8]. MSCs can increase the number of CD4^+^CD25^+^FoxP3^+^ T-regulatory cells that suppress the immune response [6–8]. Susceptibility to diabetes induction and development may be related to the activity of T-regulatory cells [9] and expansion of Th17 cells [10]. MSCs are able to render T cells anergic by blocking differentiation of monocytes to DCs or by inhibiting DC maturation [6]. Through production of soluble factors, MSCs can inhibit proliferation and IgG secretion of B cells [8]. Thus, it appears that therapeutic effects and use of MSCs would be primarily based on their release of trophic and immunomodulatory factors [8, 9] (supporting information Fig. 2).

Previous studies have shown that MSCs are able to differentiate into several cell types, including cardiomyocytes, vascular endothelial cells, neurons, hepatocytes, epithelial cells, and adipocytes, making them a potentially important source for the treatment of debilitating human diseases [11]. Such multipotent differentiation characteristics coupled to their capacity for self-renewal and capability for the regulation of immune responses, described MSCs as potentially new therapeutic agents for treatment of the complications of diabetes mellitus (DM) [11].

## EFFECT OF MSCS ON ISLET PATHOLOGY

The possible therapeutic effect of MSCs in diabetes is suggested by their capacity to generate insulin-producing cells (IPCs) [8, 12, 13]. These IPCs express multiple genes related to the development or function of pancreatic beta cells, including high expression of pancreatic and duodenal homeobox 1, insulin, and glucagon [12] and were able to release insulin in a glucose-dependent manner that led to amelioration of diabetic conditions in streptozotocin (STZ)-treated nude mice [12, 13]. Several lines of evidence suggest that in vivo hyperglycemia is an important factor for bone marrow-derived MSCs differentiation into IPCs capable of normalizing hyperglycemia in a diabetic animal model [14, 15], including animals with chronic hyperglycemia and cardiomyopathy.

## MSC TREATMENT OF DIABETIC CARDIOMYOPATHY

Development of ventricular dysfunction in patients with DM in the absence of coronary artery disease, valvular heart disease, or hypertension is defined as diabetic cardiomyopathy (DCM) [16]. Chronic hyperglycemia is responsible for myocardial remodeling and is a central feature in the progression of DCM, which is characterized by hypertrophy and apoptosis of cardiomyocytes and alterations in the quantity and composition of the extracellular matrix (ECM) resulting in increased collagen deposition [17]. An additional feature that contributes to the pathogenesis of DCM is the activity of matrix metalloproteinase (MMP)-2 and MMP-9 [18, 19]. The diabetic myocardium is characterized by decreased activity of MMP-2, leading to increased collagen accumulation, and increased activity of the proapoptotic factor MMP-9, which is responsible for apoptosis of endothelial cells, reduction of capillary density, and poor myocardial perfusion [18, 19]. Microcirculatory defects, necrosis and apoptosis of cardiomyocytes, and interstitial fibrosis are the main pathological characteristics of DCM [16, 19].

**Figure 1:**
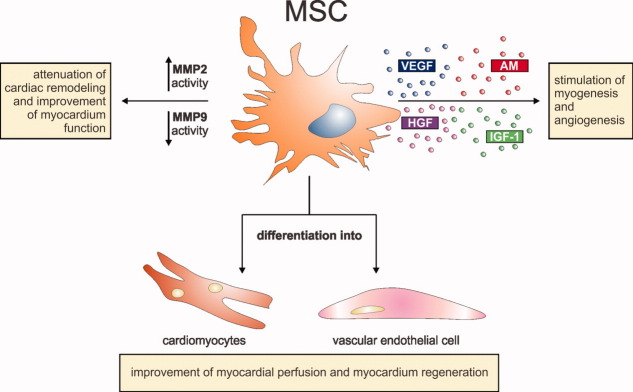
Effects of transplanted MSCs on diabetic cardiomyopathy. (a) MSCs increase the activity of MMP-2 and decrease the activity of MMP-9 and attenuate cardiac remodeling. (b) MSCs produce VEGF, IGF-1, AM, HGF and stimulate myogenesis and angiogenesis in damaged myocardium. (c) Through differentiation into cardiomyocytes and vascular endothelial cells, MSCs improve myocardial perfusion and myocardium regeneration. Abbreviations: AM, adrenomedullin; HGF, hepatocyte growth factor; IGF-1, insulin-like growth factor-1; MMP, matrix metalloproteinase; MSC, mesenchymal stem cell; VEGF, vascular endothelial growth factor.

MSCs can also induce myogenesis and angiogenesis by releasing different angiogenic, mitogenic, and antiapoptotic factors including vascular endothelial growth factor (VEGF), insulin-like growth factor-1 (IGF-1), adrenomedullin (AM), and hepatocyte growth factor (HGF) [20]. This was demonstrated using a rat model of DCM [20] wherein intravenous (i.v.) administration of bone marrow-derived rat MSCs improved cardiac function of treated animals. Transplanted MSCs differentiated into cardiomyocytes and improved myogenesis and angiogenesis [20]. In addition, MMP-2 activity significantly increases and MMP-9 activity decreases after MSCs transplantation [20]. This phenomenon increases myocardial arteriolar density and decreases collagen volume resulting in attenuation of cardiac remodeling and improved myocardial function [20] (Fig. 1).

Improvement in cardiac function following MSC therapy may also be attributed to the release of MSC-derived paracrine factors capable of cardioprotection. These factors include secreted frizzled-related protein 2, Bcl-2, heat shock protein 20, hypoxia-regulated heme oxygenase-1, hypoxic Akt-regulated stem cell factor, VEGF, HGF, AM, and stromal-derived factor [21]. A growing body of evidence strongly suggests that these factors affect remodeling, regeneration, and neovascularization leading to the improvement of myocardium contractility and viability, ameliorating consequences of infarction [21–26]. Double-blind, placebo-controlled trials showed that i.v. autologous MSCs transplantation increased left ventricular ejection fraction, reduced episodes of ventricular tachycardia, and led to reverse remodeling in postinfarction patients reducing the mortality rate in patients with ischemic stroke [22, 23].

Similar effects and MSC-derived proangiogenic factors have also been implicated in the therapy of diabetic limb ischemia [24, 25]. VEGF and hypoxia-inducible factor are responsible for restoring blood flow and vasculogenesis in the ischemic hindlimb of type II diabetic (db[−]/db[−]) mice [24] or for improvement of arterial perfusion in type 1 diabetic patients with bilateral upper extremity digital gangrene [25].

## MSC TRANSPLANTATION IMPROVES CARDIAC FUNCTION IN DIABETIC ANIMALS

Bradycardia, decrease in left ventricular developed pressure, decrease of contractility index and increase in arterial pressure occur in diabetic animals [26, 27]. Bradycardia, accompanied by a remarkable decrease in left ventricular developed pressure, happens because of cardiac sympathetic nerve impairment [27]. In addition to impairment of the sympathetic innervation of the heart and low levels of cardiomyocyte cytosolic Ca^2+^, cardiac remodeling and insulin deficiency are also responsible for an impairment of cardiac function in diabetes [26, 27]. Insulin signaling plays an important role in regulating myosin isoform expression and expression of glucose transporter 1 in the myocardium [28]. It is accepted that insulin can improve cardiac function by its inotropic effect of reducing blood glucose levels to prevent further myocardial remodeling [28].

MSCs treatment of diabetic rats results in a significant increase in heart rate, left ventricular pressure, contractility index, and notable reduction of systolic blood pressure [29]. The improvement in cardiac condition can be explained by differentiation of MSCs into IPCs [12, 13], cardiomyocytes and vascular endothelial cells [3] and also by the immunomodulation ability of MSCs [6–8]. Significant increase of serum insulin levels leads to endothelial cell protection, and this is accompanied with enhanced myogenesis, angiogenesis, and attenuation of cardiac remodeling, all of which are crucial for the improvement of cardiac function in diabetic animals [20, 21, 28, 29].

## MSC THERAPY OF DIABETIC NEPHROPATHY

MSCs administration can prevent and treat diabetic nephropathy, which is a complication of DM and is defined as progressive kidney disease caused by angiopathy of the capillaries supplying the kidney glomeruli [30]. MSCs have been used for the treatment of diabetic nephropathy in nonobese diabetic/severely compromised immunodeficient (NOD/SCID) and C57 black 6 (C57/BL6) mice, which succumb to DM after application of multiple low doses of STZ [30, 31]. About 30–60 days after STZ injection, kidneys of treated mice showed the presence of abnormal glomeruli characterized by increased deposits of ECM protein in the mesangium, hyalinosis, and increased number of macrophages in the glomeruli [30, 31].

Data obtained from studies using NOD/SCID mice transplanted with human MSCs (hMSCs) and C57Bl/6 mice that received murine MSCs indicate that injected MSCs engraft in damaged kidneys, differentiate into renal cells, and regulate the immune response resulting in an efficient treatment of diabetic nephropathy [30, 31]. Additionally, the small percentage of hMSCs in the transplanted kidneys differentiated into endothelial cells as evidenced by de novo expression of CD31 [31]. The result of systemic administration of MSCs in diabetic mice was improvement of kidney function and regeneration of glomerular structure [30, 31] as MSCs are able to reconstitute necrotic segments of diabetic kidneys [32]. However, it is not clear whether MSCs can propagate after engraftment in the kidney [30, 31]. One month after MSC treatment, only a few hMSCs were detected in kidneys, suggesting that they were unable to proliferate [31] so an alternative scenario for improvement of kidney function could be the ability of MSCs to scavenge cytotoxic molecules or to promote neovascularization [21–26]. In addition, successful MSC treatment of diabetic nephropathy could be explained by MSCs competence to differentiate into insulin-producing beta cells followed by decrease of glycemia and glycosuria, factors important for damaging renal cells [30]. Taken together, these data indicate that MSC transplantation prevents the pathological changes in the glomeruli and enhances their regeneration resulting in improved kidney function in diabetic animals.

## MSC TREATMENT OF DIABETIC POLYNEUROPATHY

Diabetic polyneuropathy (DPN), the most common complication of DM, is characterized by damage to nerve fibers [33]. Spontaneous pain, hyperalgesia, and diminished sensation are symptoms of DPN [33]. The central features in the development and progression of DPN are neural cell degeneration and decreased nerve blood flow (NBF) [33].

**Figure 2:**
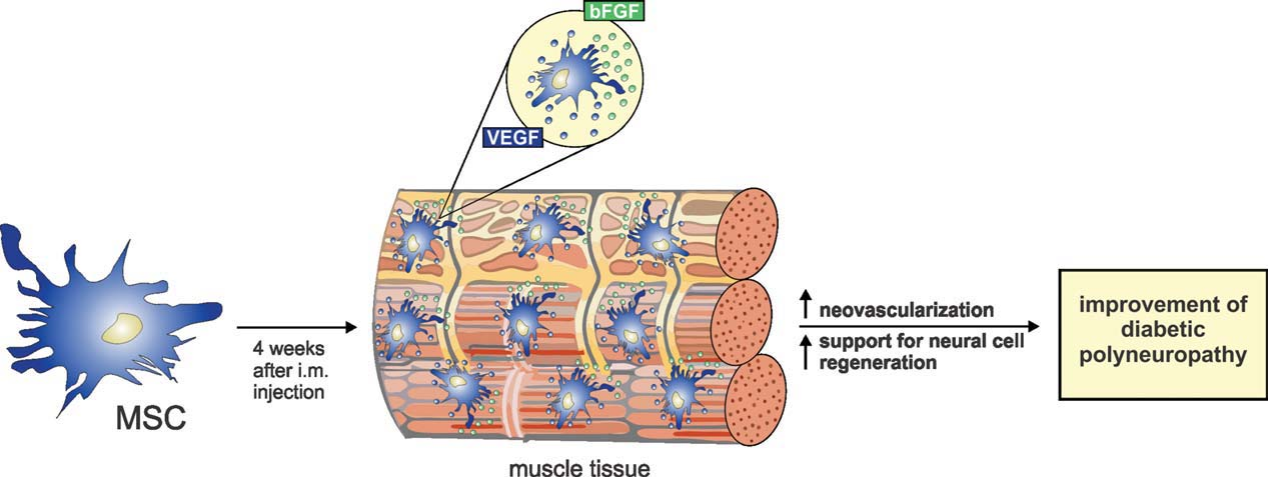
Effects of MSCs treatment on diabetic polyneuropathy. Four weeks after intramuscular injection, MSCs settled in the gap between muscle fibers, through production of bFGF and VEGF, induces neovascularization and support regeneration of neural cells that results with improvement of diabetic polyneuropathy. Abbreviations: bFGF, basic fibroblast growth factor; MSC, mesenchymal stem cell; VEGF, vascular endothelial growth factor.

Previous studies have shown that angiogenic cytokines such as basic fibroblast growth factor (bFGF) and VEGF could be useful for the treatment of DPN [21, 34, 35]. It was shown in diabetic rats that MSCs, because of their ability to secrete bFGF and VEGF [21], could be used as a new and effective therapeutic agent for the treatment of DPN [34, 35]. Four weeks after intramuscular injection, MSCs settled in the gap between muscle fibers, produced bFGF and VEGF and led to increase in the ratio of capillaries to muscle fibers that was followed by improvement of hyperalgesia, and a corresponding functional improvement of neural fibers, delayed motor nerve conduction velocity, reduced sciatic NBF, and decreased axonal circularity at the site of transplantation [35] (Fig. 2).

Although several studies [1, 3, 11] have suggested that MSCs have the capacity to differentiate into neural cells, such as astrocytes, oligodendrocytes, and Schwann cells, this was not noted after MSCs transplantation in diabetic rats [35]. After intramuscular injection, MSCs remained at the transplant sites. They did not have any systemic effects and did not differentiate into neural cells, which suggest that systemic MSCs injection could be a better way for the improvement of all affected nerve fibers [35]. However, by releasing paracrine factors and through differentiation into photoreceptor and glial-like cells in the retina, transplanted MSCs improved the integrity of the blood-retinal barrier thus ameliorating diabetic retinopathy in STZ diabetic rats [36].

## MSC TREATMENT OF DIABETIC WOUNDS

Prolonged and incomplete wound healing, caused by reduced production of growth factors, impaired angiogenesis, and compromised formation of collagen matrixes, was observed as a complication of DM [37, 38]. The characteristics of diabetic wounds are poor neovascularization, presence of abundant inflammatory infiltrates mostly containing polymorphonuclear cells, and foci of necrotic tissue composed of neutrophils [37]. Disturbances in collagen metabolism and compromised production and functionality of growth factors such as transforming growth factor β (TGF-β), epidermal growth factor (EGF), VEGF, platelet-derived growth factor (PDGF), and keratinocyte growth factor (KGF) are the main factors responsible for the pathogenesis of poor wound healing [37]. Systemic and local administration of bone marrow-derived MSCs improves healing of diabetic wounds in rats and mice [39]. After i.v. injection of MSCs, diabetic wounds showed significantly increased collagen levels followed by increased wound-breaking strength [39]. The increased production of collagen, the major component of ECM crucial for strength, integrity, and structure of normal tissues and important for repairing tissue defects created by injuries, was noticed after MSC administration [39]. MSC injection resulted in moderate (TGF-β, KGF) or significant (EGF, PDGF, and VEGF) increase in the production of growth factors involved in the repair of injured tissue that was crucial for successful diabetic wound healing [39]. These factors stimulated cell adhesion at the site of injury and induced cells to secrete more chemokines resulting in neovascularization and formation of inflammation infiltrate, containing predominantly mononuclear cells, without tissue necrosis [21, 39]. Beside these paracrine effects, MSCs can help to improve diabetic wounds through their differentiation ability [40] and ability to regenerate damaged epithelium through differentiation and fusion [41]. In diabetic mice, some of the MSCs transplanted in the wound, coexpressed cytokeratin, whereas others formed sweat or sebaceous gland-like structures of the skin [40] (supporting information Fig. 3).

Although MSCs were not found in the vascular structures of diabetic wounds, it was documented that, after MSC treatment, there was enhanced capillary density in those, suggesting that MSCs promoted angiogenesis that was very important for successful healing [40, 41]. In diabetic wounds, MSCs settled predominantly in the newly formed dermis, and to a lesser extent in the epidermis, however, none were detected in the undamaged skin [40–42]. MSCs have already shown efficacy in the treatment of foot ulcerations in diabetic patients [43]. Autologous biografts composed of skin fibroblasts seeded on biodegradable collagen membranes in combination with autologous MSCs, derived from the patient's bone marrow, were successfully used for closing and healing diabetic foot ulcerations [43]. There is a difference in the efficacy between systemic and local MSCs therapy for diabetic wound healing [40, 43]. For example, better effects are noticed after local administration of MSCs, possibly because of the presence of arterial-venous shunts in diabetic skin, which may complicate migration of systemically injected MSCs to the wounds [40].

## THE LIMITATIONS OF THERAPEUTIC USE OF MSC

There are several problems that limit the therapeutic use of MSCs at present [44–46]. Poor engraftment and limited differentiation under in vivo conditions are major obstacles for efficient therapeutic use of MSCs [44]. The frequency of spontaneous differentiation of MSCs in the host tissue is extremely rare, therefore, therapeutic use of MSCs depends on the ability to control their in vivo differentiation into functional cells with high efficiency and purity [44]. An additional limitation is the potential of MSCs to differentiate into unwanted mesenchymal lineages [45], which could impair their therapeutic use. There are data suggesting the restriction of such unwanted differentiation by a variety of factors [46], however, this problem is still largely unsolved because the precise roles of factors that could be responsible for the fate of MSCs after their administration are not completely understood [6, 45]. Additional limitations are possible malignant transformation and cytogenetic aberrations of MSCs. This was observed after in vitro growth of murine MSCs derived from the bone marrow of Bagg albino (BALB/c) and C57BL/6 mice [44]. However, malignant transformation of transplanted hMSCs has not yet been noted [6].

## CONCLUSION

Because of their immunomodulatory ability, self-renewal, and differentiation capacity, MSCs are expected to become promising therapeutic agents for improvement of cardiac function and treatment of DCM, nephropathy, DPN, and wounds in diabetic patients [20, 30–32, 36, 39, 40]. Among stem cells, MSCs have several advantages for therapeutic use such as ability to migrate to the sites of tissue injury, strong immunosuppressive effects [6–8], better safety after infusion of allogeneic MSCs [22, 23], and lack of ethical issues, such as those related to the application of human embryonic stem cells [2]. However, there are several outstanding problems including potential risk of malignant transformation of MSCs, unwanted mesenchymal lineages differentiation, and suboptimal targeted differentiation, which should be addressed before MSCs can be defined as a novel and efficient therapeutic agent in the treatment of the complications of DM.
