# Supplementary figures and images for: Concise Review: Mesenchymal Stem Cell Treatment of the Complications of Diabetes Mellitus
Source: Stem Cells. 2010 Nov 9;29(1):5–10. doi: 10.1002/stem.556 (PMC3059410; doi:10.1002/stem.556)

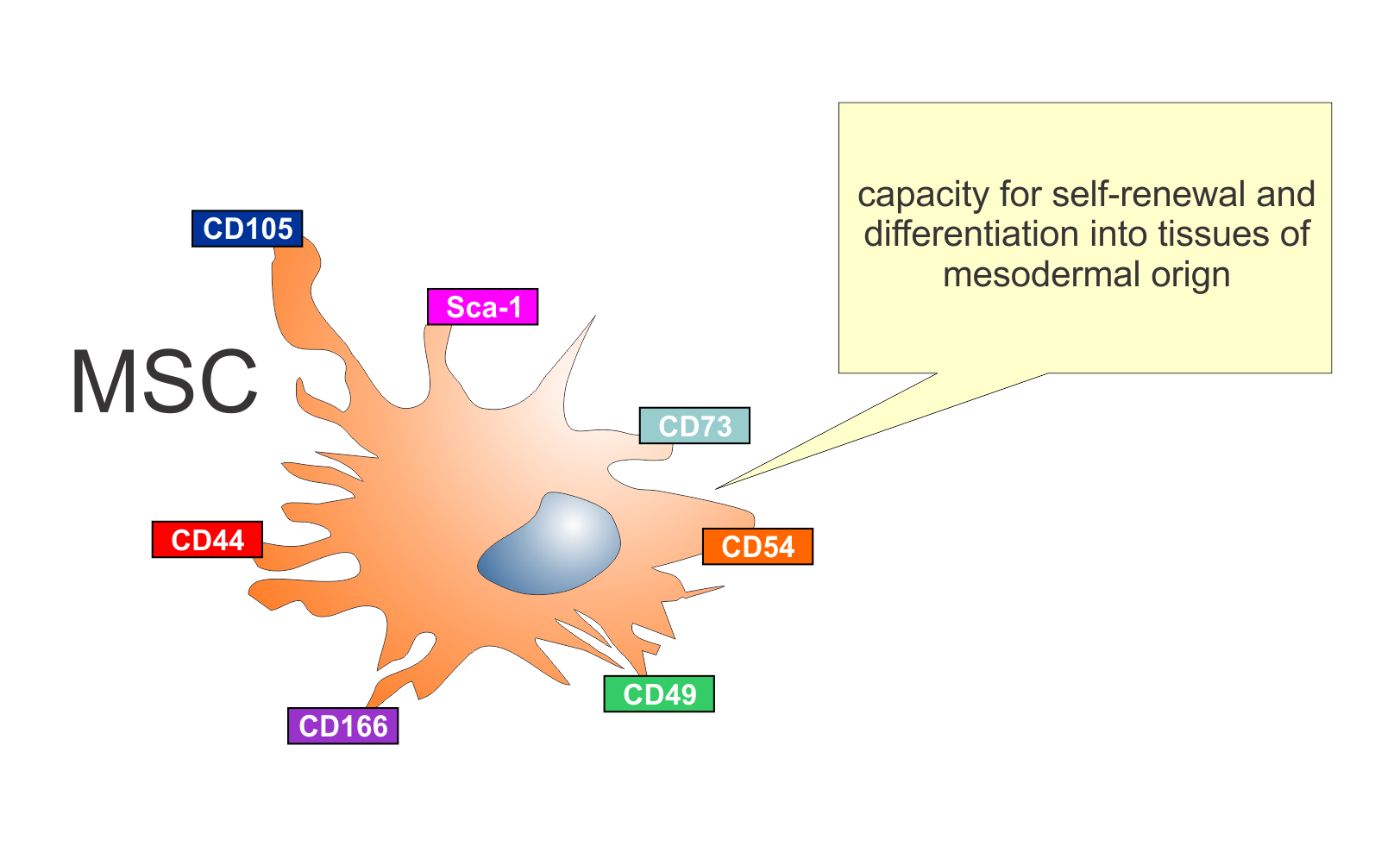

Supplement: Supplementary file 1 [file stem0029-0005-SD1.tif]

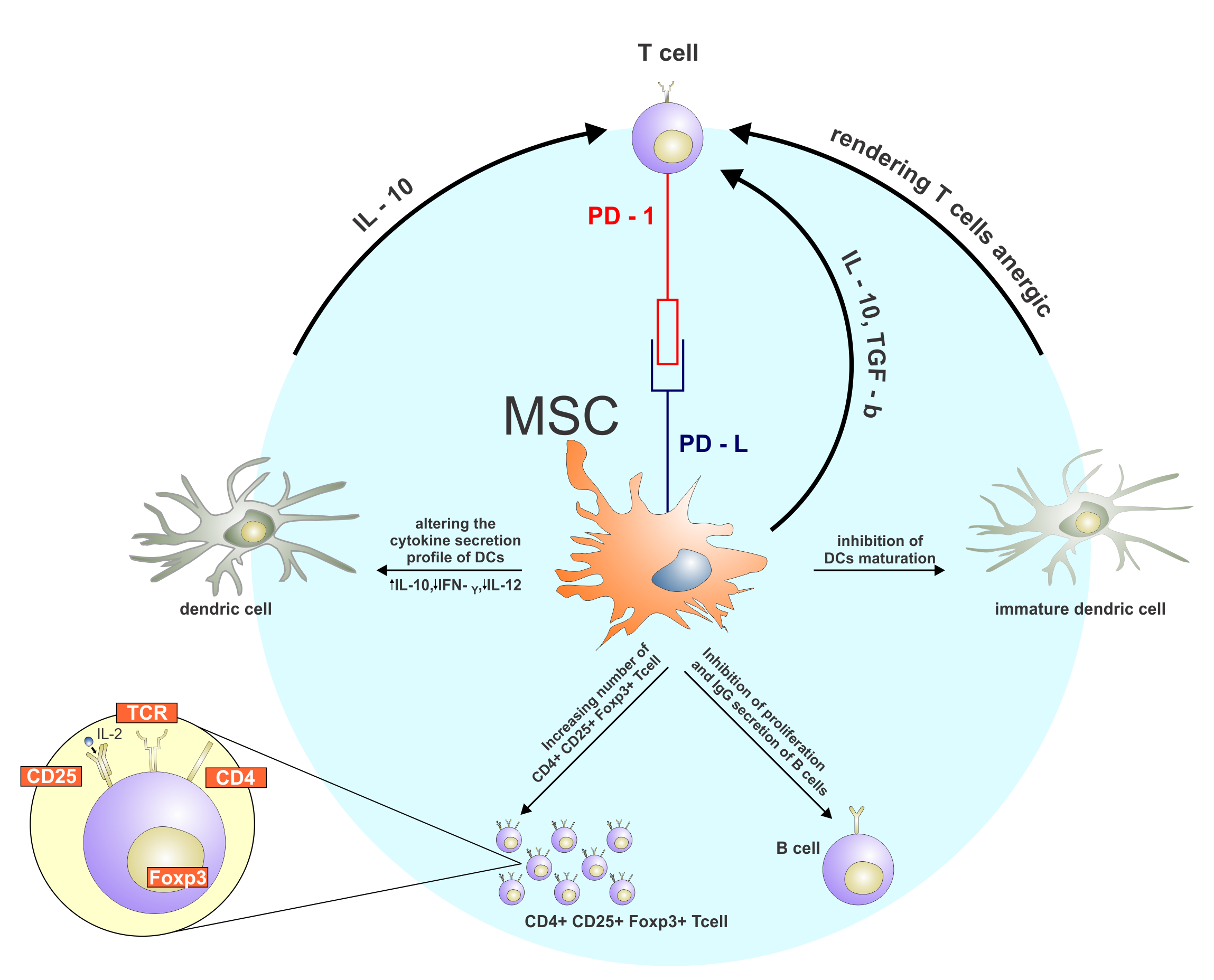

Supplement: Supplementary file 2 [file stem0029-0005-SD2.tif]

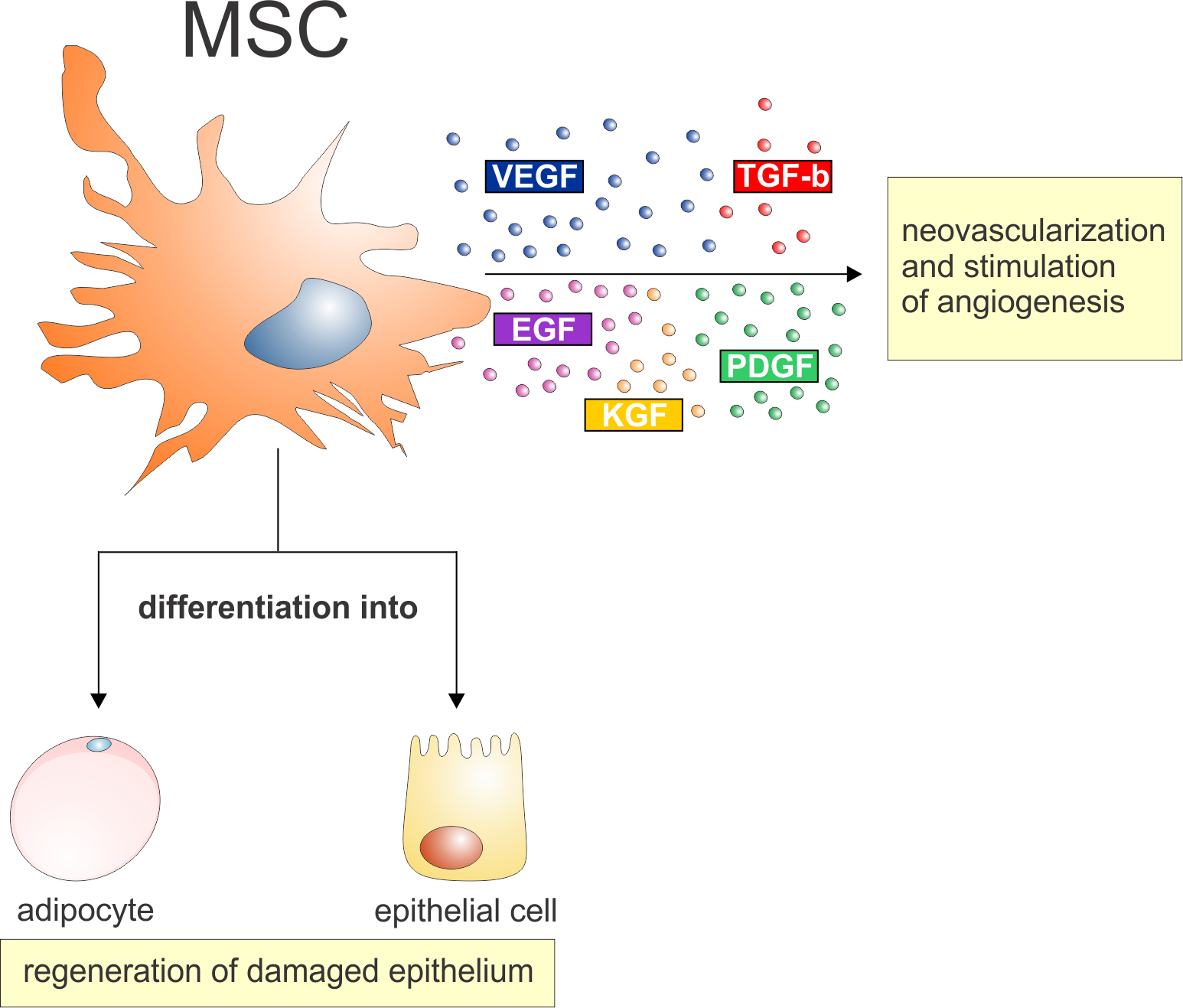

Supplement: Supplementary file 3 [file stem0029-0005-SD3.tif]
